# Supplementary material for: Protein-based condensation mechanisms drive the assembly of RNA-rich P granules
Source: eLife. 2021 Jun 9;10:e63698. doi: 10.7554/eLife.63698 (PMC8238508; doi:10.7554/eLife.63698)
Supplement: Supplementary file 1. [file elife-63698-supp1.docx]

| **Strain** | **Genotype** | **Description** | **Derived from** | **Guide RNA sequence(s)** | **Repair Template** | | **Publication** |
| --- | --- | --- | --- | --- | --- | --- | --- |
| JH3477 | *meg-3(ax3051) meg-4(ax3052)* | MEG-3::OLLAS  *meg-4* deletion | JH3374 | tctgcccaggaacttgtaac, ggagtttggcagatcacatg | gttgcaggtatgagttcttcaaagctttcctcatgtgggaagtttgtccagagcagagg aacgggtagttttctattgttatcaggactgctgc | | Smith et al., 2016 |
| JH3479 | *meg-3(ax3056) meg-4(ax3052)* | MEG-3_IDR_::OLLAS  *meg-4* deletion | JH3422 | same as JH3477 | same as JH3477 | | Smith et al., 2016 |
| JH3517 | *meg-3(ax4500) meg-4(ax2080)* | MEG-3_698_::OLLAS  MEG-4::3xFLAG | JH3374 | tgaaagcttgacagcattcc,tcagtacaatcattgatctc | caccacctcgcatttctgaaagcttgacagcattccaatccggattcgccaacgagctcggaccacgtctcatgggaaagtgattgtaccaatttatatctattacttgtagactata | | This study |
| JH3630 | *meg-3(ax4500) meg-4(ax)* | MEG-3_698_::OLLAS  *meg-4* deletion | JH3517 | same as JH3477 | same as JH3477 | | This study |
| JH3632 | *meg-3(4501) meg-4(ax3052)* | MEG-3(HMGL deletion)::OLLAS  *meg-4* deletion | JH3477 | gtcaagctttcagaaatgcg, atccaatcttggaattgtct | ctcaagatccagcttcaacctcgccaccacctcgcacaattccaagattggatggtccttatgccgatgg | | This study |
| JH3861 | *meg-3(4502) meg-4(ax3052)* | MEG-3_HMGL-_::OLLAS  *meg-4* deletion | JH3632 | tccaatcttggaattgtgcg | ctcaagatccagcttcaacctcgccaccacctcgcatttctgaaagcttgacagcatttttggaggcgcaacaggatgccaacgacgctattgatactaacgccaaagaaaagacacaactcctgaaagtgaatttggctattcacgggatgtcacctgaaagatggctgtacttgaattatttttgcaccgagacaattccaagattggatggtccttatgccgatgg | | This study |
| JH3420 | *meg-3(ax4503) meg-4(ax2080)* | MEG-3_Cterm_::OLLAS  MEG-4::3xFLAG | JH3374 | tcctcaaaaccttacccaag, tcagatcaatcggaacaatg | gatttttgcaggtatgagctcctcaaaaccttacccaaatgtggatgtaaagagaacaccttcctcgtcaatc | | This study |
| JH3553 | *meg-3(ax4503) meg-4(ax4504)* | MEG-3_Cterm_::OLLAS  *meg-4* deletion | JH3420 | same as JH3477 | same as JH3477 | | This study |
| JH3475 | *meg-3(ax3055) meg-4(ax3052)* | *meg-3* deletion *meg-4* deletion | JH3439 | same as JH3477 | same as JH3477 | | Smith et al., 2016 |
| **Supplementary file 1:** *C. elegans* strains used in this study, generated by CRISPR/Cas9 genome editing | | | | | |  |  |
